# Tumor regression efficacy of *Punica granatum L* leaf extract against K562 leukemia cancer cells

**DOI:** 10.6026/97320630019536

**Published:** 2023-05-31

**Authors:** Poojitha B Sridhara Setty, Gopinath SM

**Affiliations:** 1 Department of Studies in Biotechnology, Davangere University, Davangere - 577 007, Karnataka, India

**Keywords:** Leukemia cancer cells, Punica granatum, cell cycle arrest, apoptosis

## Abstract

It is of interest to describe the extraction and isolation of ethyl heneicosanoate (EHO) from Punica granatum. L plant leaves. Further, the structure of isolated molecule, EHO was confirmed through various spectroscopic tools viz., infrared (FT-IR), proton NMR and mass spectroscopy. The tumor regression potential of EHO was evaluated against K562 leukemia cancer cells. A significant reduction in the viability and metabolic activity was obtained in vitro for K562 cell lines when treated with EHO molecule. Further, the results depicted that the activities of EHO is directly related to the alkyl chain length. The xenograft model of mice illustrated a lower propensity of tumor growth in the group receiving EHO molecule, compared with the group receiving Bortezomib (positive control). The obtained results suggest that the animals treated with Bortezomib showed a tumor growth inhibition up to 77.51 ± 3.74 whereas, the EHO showed the inhibition up to 23.37 ± 25.44 and 40.64 ± 16.45 % at 200 mg/kg and 400 mg/kg respectively. Moreover, the results indicate that the activity of EHO in the induction of apoptosis in induced leukemia cancer.

## Background:

Cancer is one of the most detrimental diseases in the world since long time. Several million people are diagnosed for this disease each year, resulting in deaths in about 25-30% of the cases [1]. The major reasons for the high rate of morbidity are an old age population and the addiction of unhealthy eating habits, smoking, low physical activity, and obesity [2, 3]. Novel therapies for cancer have been developed so far that include various surgery for primary disease, resection of metastatic disease affecting, radiotherapy, chemotherapy and so on [4, 5]. Moreover, the commercial anticancer drugs that are available would cause many other side effects and complications [6, 7]. Due to the this, there is an emergency in availing the novel drugs that are less toxic and cost-effective that would both inhibit the cancer growth and prevent further relapses. Apoptosis is a cell death process characterized by morphological and biochemical features occurring at different stages. Once triggered, apoptosis proceeds with different kinetics depending on cell types and culminates with cell disruption and formation of apoptotic bodies. A critical stage of apoptosis involves the acquisition of surface changes by dying cells that eventually results in the recognition and the uptake of these cells by phagocytes. Different changes on the surface of apoptotic cells such as the expression of thrombospondin binding sites, loss of sialic acid residues and exposure of a phospholipid like phosphatidylserine (PS) were previously described [8]. Phospholipids are asymmetrically distributed between inner and outer leaflets of the plasma membrane with phosphatidylcholine and sphingomyelin exposed on the external leaflet of the lipid bilayer, and phosphatidylserine predominantly observed on the inner surface facing the cytosol [9]. Exposure of PS on the external surface of the cell membrane has been reported for activated platelets and senescent erythrocytes. Recently, it was shown that cells undergoing apoptosis break up the phospholipid asymmetry of their plasma membrane and expose PS which is translocated to the outer layer of the membrane[10,[11]] . This occurs in the early phases of apoptotic cell death during which the cell membrane remains intact. This PS exposure may represent a hallmark (early and widespread) in detecting dying cells. Therefore, it is [11] of interest to document the tumor regression efficacy of *Punica granatum L* leaf extract against K562 leukemia cancer cells.

##  Material and Methods:

The chemicals and reagents used in this study were obtained from Sigma and Merck chemical industries, India. Thin layer chromatography purification of EHO was carried out using ore-coated aluminum plate as stationary phase and CHCl3: MeOH (9:1) as mobile phase.

## Extraction and isolation of ethyl heneicosanoate:

20 g of dried leaf powder of Punica granatum. L plant leaves was dissolved in 100 mL of Methanol in a 500 mL beaker with aluminum foil covered on it. Then the beaker was kept on hot water bath at 50°C for 4 hours. After incubation period the extract was filtered using Whatmann filter paper. Residue present over the filter paper was discarded. Thus, obtained filtrate was reduced under pressure to get a semisolid form. This crude extract is then subjected to HPLC technique for further purification. ACN: Water (HPLC grade) was used as mobile phase (60:40%) with a flow rate of 1 mL/min. The absorption of fractions was measured at 290 nm. The methanol fraction is taken for all the further studies as its purity is 100% as per HPLC analysis. This fraction is further analyzed for LCMS, FTIR and NMR for structure elucidation.

## Cell cycle studies:

1 X 106 cells were seeded and cultured for 24 h in a 6-well plate containing 2 ml of medium. The cells were then treated with the desired concentration of sample and incubated for an additional 24 h [5]. Cells were harvested, centrifuged, washed, fixed, stored at 4°C overnight, centrifuged, washed twice, resuspended in envelope fluid containing propidium iodide and RNaseA, incubated for 15 min, and percentages of cells at different stages of the cell cycle determined using a FACS caliper.

## Flow Cytometric analysis:

The cell cycle distribution was investigated by flow cytometric studies as previously described [6]. Cells were seeded in 6-well plates and treated with EHO media or molecules for 48 h, then washed with PBS, fixed in ethanol, stored at -20°C, and incubated with propidium iodide staining buffer. Cells were washed with ice-cold PBS, resuspended in buffer containing Annexin V-FITC and PI, and incubated at 37°C for 30 min. Apoptotic cells were analyzed by flow cytometry [7].

## FITC staining of K562cells by flow cytometry:

Plate 1 X 106 cells per well to a 6-well plate using Dulbecco's Modified Eagle Medium (DMEM). After 18 h, the wells floated and replaced with the new culture medium. Treated cells were induced apoptosis and incubated for 24 h. Cells were collected in Ria flasks and resuspended in 1X binding buffer. 500 L of the cell suspension was divided and 10 L of PI and Annexin V were added. Incubated for 15 min at AT.

## Results and Discussion:

## Spectroscopic analysis

**Figure 1:**
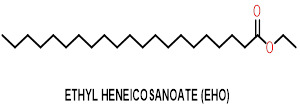
Molecular structure of isolate Ethyl Heneicosanoate (EHO).

The EHO molecule has significant absorption bands at 3250, 1705 and 1613 cm-1 due to the hydroxyl, carbonyl and C=C groups. LCMS and 1H NMR confirm the molecular structure of the EHO compound, which is shown in Figure 1.

## Effect of EHO on K562 cells growth:

K562 cells are well-known human chronic myeloid leukemia CML). In this study, we first examined the effects of the EHO molecule on K562 cancer cell viability. Cells were treated with vehicle or EHO for 24h, then cell viability was determined by MTT assay. As shown in Figure 3, EHO significantly inhibited the viability of K562 cells in a time and dose-dependent manner.

## Effects of EHO on cell cycle distribution and apoptosis of K562 cells:

**Figure 2:**
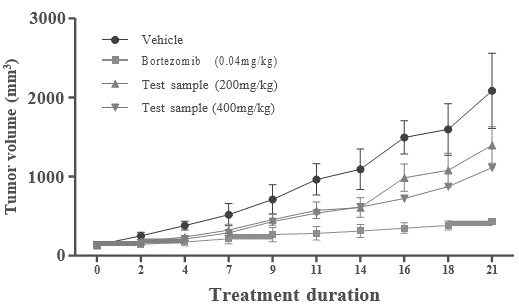
Effects of EHO on the cell growth of K562 cells.

**Figure 3:**
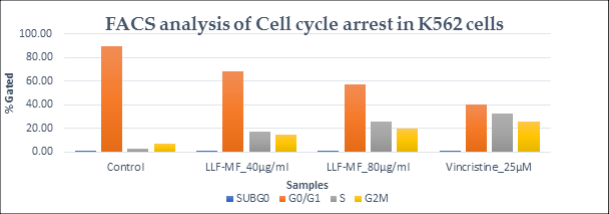
Flow cytometry analysis of cell cycle arrest in K562 cells.

**Figure 4:**
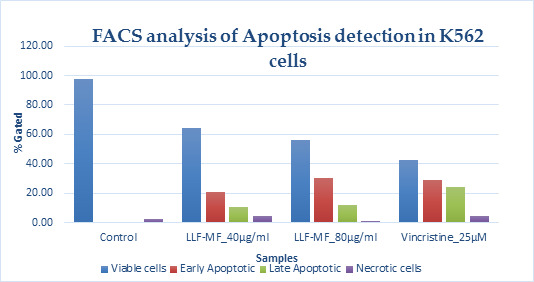
Flow cytometry analysis of apoptosis detection in K562 cells.

Furthermore, we investigated whether EHO can be regulated during cell cycle progression. As shown in Figure 2, EHO induced a significant increase in the number of cells in the G1 phase, which was complemented by a decrease in cell distribution in the S phases. EHO did not induce a major change in phase sub-G1 (dead cell population). We further investigated the apoptosis efficiency of EHO on K562 cells. As shown in Figure 3 and Figure 4, the percentage of early or late apoptotic cells did not change significantly in EHO-treated cells compared with the control group. On the other hand, the obtained results show that EHO molecule suppresses cell growth by cell cycle arrest in G1 phase but does not induce significant cell death in K562 cells.

## FACS analysis:

**Figure 5:**
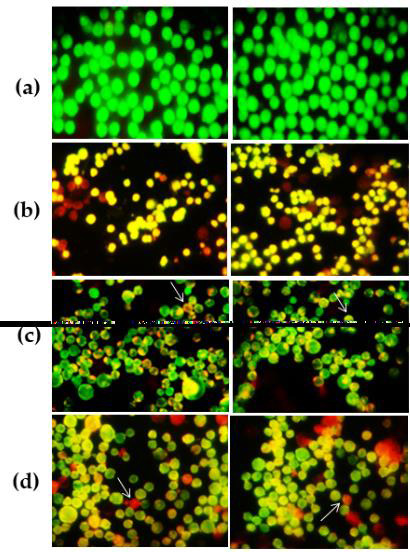
Morphological identification and annexin V of apoptotic K562 cells after EHO treatment. (a) Control K562 cells, (b) Standard Vincristine (25 µM), (c) EHO molecule (40 µg/ml) and (d) EHO molecule (80 µg/ml).

Regarding FACS analysis, we observed an increase in pro-apoptotic CASPASE 3 after treatment compared with controls (Table 1, Table 2, Figure 3, Figure 4 and Figure 5). Caspase is known to be involved in apoptosis, and thus caspase activation leads to DNA damage and fragmentation [8, 9,10]. In a recent study, the scientists reported that exosomes released by UC-MSC sensitized K562 cells to imatinib-induced apoptosis through caspase activation.

## EHO alter the morphology of K562 cells

Morphology of EHO-treated K562 cancer cells showed mild to moderate morphological changes on the cell surface at 24, 48 and 72 h by acridine orange/ethidium bromide (AO/EB). During co-culture of K562 cancer cells, [11] EHO-glued cells were further established and further demonstrated various changes in their morphological characteristics, with a few showing shrinkages of cells, while most were rounded compared to the control (Figure 4 and Figure 5). EHO-treated K562 cells often showed cell hypertrophy that was subsequently associated with membrane damage leading to cell death (Figure 4 and Figure 5).

Cells were later collected and stained with AO/EB and visualized under fluorescence microscopy. Viable cells possess a homogeneous green stain, while apoptotic cells were orange-red and further lead an evidence of cell shrinkage and nuclear condensation.

## Conclusion:

The results showed that the EHO molecule inhibited the growth of K562 cells, mainly by inducing cell cycle arrest and leading to apoptosis. Notably, the results also demonstrate that the EHO molecule exhibits increased inhibitory activity at higher concentrations. However, further study of proteomics and metabolism is needed to understand the mechanism involved in cancer cell suppression.

## Figures and Tables

**Table 1:** Fluorescence activated cell sorting (FACS) analysis of cell cycle arrest in K562 cells.

**Samples**	**SUBG_0_**	**G_0_/G_1_**	**S**	**G_2_M**
Control	0.59	89.27	2.64	7.35
EHO at 40 µg/ml	0.16	68.24	17.08	14.96
EHO at 80 µg/ml	0.05	57	25.73	19.75
Standard Vincristine at 25 µM	0.05	40.04	32.94	25.45

**Table 2:** Flow cytometry analysis of apoptosis detection of K562cells

**Sample**	**Viable cells**	**Early Apoptotic**	**Late Apoptotic**	**Necrotic cells**
Control	97.52	0.03	0.21	2.24
EHO at 40 µg/ml	64.5	20.79	10.5	4.21
EHO at 80 µg/ml	56.47	30.41	12.24	0.88
Standard Vincristine at 25µM	42.88	28.79	23.91	4.42
